# Assessment of Skin Phenotype Representation in a Popular Medical Licensing Educational Resource

**DOI:** 10.1001/jamanetworkopen.2020.33164

**Published:** 2021-01-12

**Authors:** Jessica P. Cerdeña, Rohit Jaswaney, Marie V. Plaisime, Lundy Braun

**Affiliations:** 1 Yale School of Medicine, Yale University, New Haven, Connecticut; 2 Department of Anthropology, Yale University, New Haven, Connecticut; 3 New York Medical College, Valhalla, New York; 4 Department of Sociology, Howard University, Washington, DC; 5 Department of Pathology and Laboratory Medicine, Warren Alpert School of Medicine, Brown University, Providence, Rhode Island; 6 Department of Africana Studies, Brown University, Providence, Rhode Island

## Abstract

This cross-sectional study examines the representation of darker skin phenotypes in tools used by students in preparation for medical licensure testing.

## Introduction

A survey of race and skin tone as depicted in medical textbooks suggests that conditions in dark skin are underrepresented.^1^ These materials often consider White skin to be the normative, which may delay diagnoses and increase morbidity and mortality for darker skinned Black, Latinx (gender-neutral term referring to people with heritage in Latin America), Asian, and indigenous patients, who compose 40% of the US population.^1,2^ Medicine requires training across a range of specialties and skin phenotypes to care for an increasingly diverse population.

The UWorld Step 2 Clinical Knowledge (CK) Question Bank (QBank) leads among study tools for medical students preparing for the second US Medical Licensing Examination (USMLE).^3^ Students often use UWorld after completing their preclinical and clinical training, reinforcing knowledge acquired both in the classroom and on the wards to aid diagnoses and management. Although medical curricula vary widely, preparation for individuals seeking their preferred residencies in the US often converges on QBanks.^3^ Skewed representation of skin phenotypes in UWorld thereby risks development of biases during a critical moment in medical education.^3^ In this analysis of skin phenotype, we hypothesized that darker skin is underrepresented in images from the UWorld Step 2 CK QBank.

## Methods

We examined 3537 questions in the UWorld Step 2 CK QBank between May 28 and August 11, 2020, recording and describing 1251 photographs or illustrations of human skin. Two of us serving as coders (J.P.C. and R.J.) independently classified these images by skin tone, collapsing the 11-point Project on Ethnicity and Race in Latin America skin color palette^4^ into light (1-4), medium (5-6), and dark (7+) categories (κ = 0.96); the coders resolved disagreements through discussion. We further categorized these images using the organization provided by UWorld and denoted images depicting any of 36 characteristic dermatologic findings.^5^ We used χ^2^ analyses to test differences among image types and systems by skin phenotype (α < .05 threshold of significance) and Poisson logistic regression to analyze variation in counts of images of hallmark dermatologic findings by skin phenotype. Testing was 2-tailed and unpaired. We entered data into Google sheets (Google LLC) and completed analyses in RStudio, version 1.1.463 (RStudio PBC). The Yale University Institutional Review Board determined this study did not constitute human subjects research. This study followed the Strengthening the Reporting of Observational Studies in Epidemiology (STROBE) reporting guideline.

## Results

In 1251 illustrations or photographs of human skin, light (n = 1127) outnumbered medium (n = 47) and dark (n = 47) skin phenotypes; the ratio of light to darker skin phenotypes was 12 to 1 (Figure, A). Darker skin appeared significantly less commonly among illustrations (n = 18) compared with photographs (n = 100) (*P* < .001) (Figure, B). Dermatology demonstrated the greatest representation of darker skin phenotypes at 17.8%; no other system surpassed 15% (Figure, C). Characteristic dermatologic findings were presented significantly less often in medium (n = 18) and dark (n = 17) skin compared with light skin (n = 213) (*P* < .001) (Figure, D). At least 1 image of light skin depicted each of the 32 characteristic findings that appeared in the QBank. No skin cancers were shown in darker skin.

**Figure.**
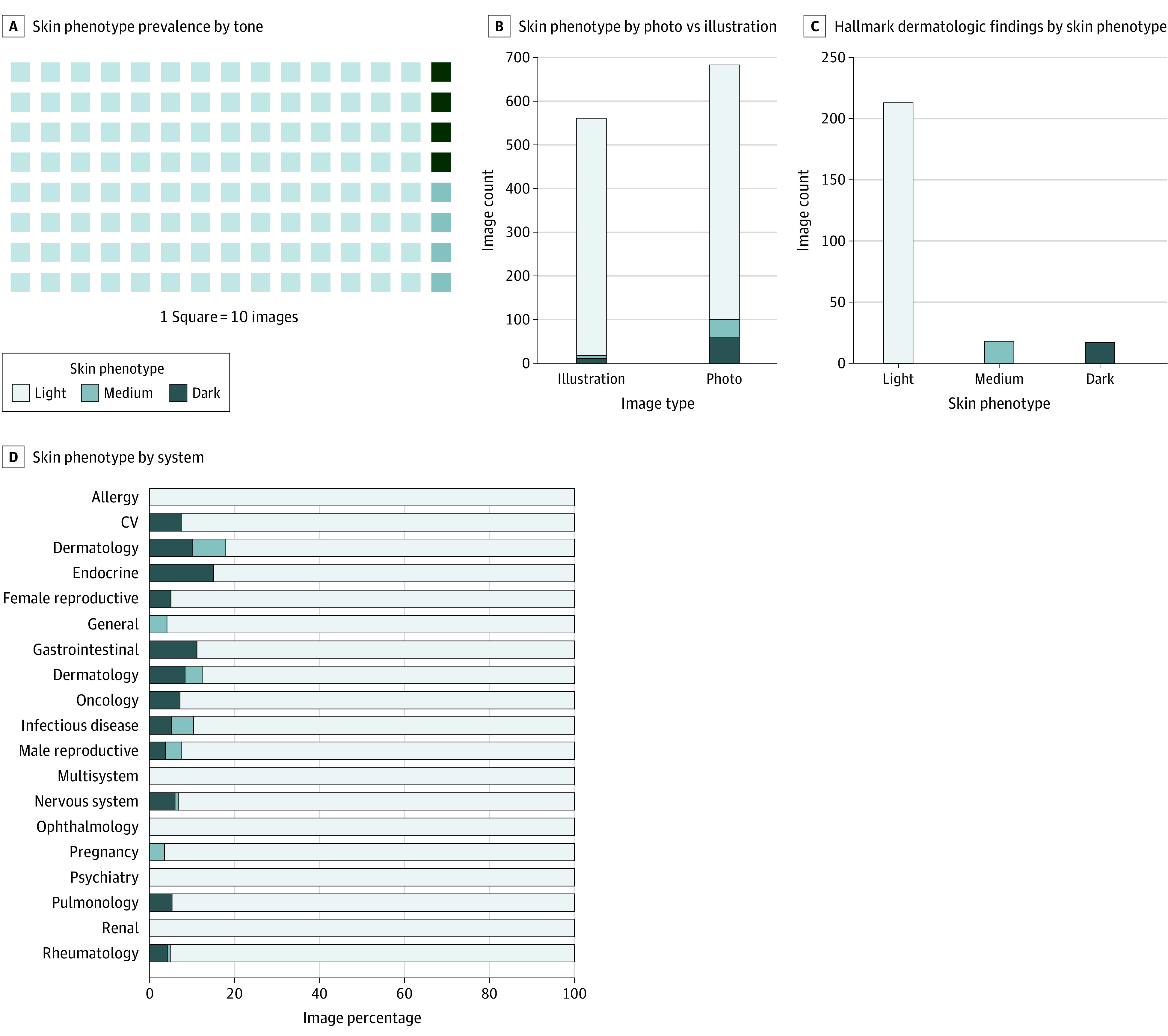
Skin Phenotype Prevalence and Differences by Photograph vs Illustration, System, and Hallmark Dermatologic Findings CV indicates cardiovascular.

## Discussion

Our findings suggest that darker skin phenotypes are underrepresented in the UWorld Step 2 QBank, a popular study tool for medical students seeking US residencies. UWorld contributors across multiple specialties should broaden representation of diverse skin phenotypes through both patient photographs and designer illustrations to ensure that clinicians are able to identify pathologic traits in darker-skinned patients. In addition, demonstration of skin cancers across a range of phenotypes may improve treatment prognosis.^6^ Our study could not assess for updates made to the QBank following the conclusion of the capture period or draw conclusions regarding the US Medical Licensing Examination Step 2 CK. Nevertheless, our analysis suggests that increasing representation of varied skin tones in medical training materials may mitigate health disparities rather than reproduce them. Partnership with the Skin of Color Society, Brown Skin Matters, and Mind the Gap organizations may support these efforts.

## References

[1] Louie P, Wilkes R Representations of race and skin tone in medical textbook imagery. Soc Sci Med. 2018;202:38-42. doi:10.1016/j.socscimed.2018.02.023 29501717

[2] United States Census Bureau US Census Bureau QuickFacts: United States. Published July 1, 2019 Accessed November 16, 2020. https://www.census.gov/quickfacts/fact/table/US/PST045219

[3] Ripp K, Braun L Race/ethnicity in medical education: an analysis of a question bank for step 1 of the United States Medical Licensing Examination. Teach Learn Med. 2017;29(2):115-122. doi:10.1080/10401334.2016.1268056 28051889

[4] Telles E. Pigmentocracies: Ethnicity, Race, and Color in Latin America. UNC Press Books; 2014.

[5] Le T, Bhushan V. First Aid for the USMLE Step 2 CK. Tenth Edition McGraw-Hill Education; 2019.

[6] Korta DZ, Saggar V, Wu TP, Sanchez M Racial differences in skin cancer awareness and surveillance practices at a public hospital dermatology clinic. J Am Acad Dermatol. 2014;70(2):312-317. doi:10.1016/j.jaad.2013.10.030 24332312

